# Exodus of Southern Medical Students

**Published:** 1860-01

**Authors:** 


EXODUS OF SOUTHERN MEDICAL STUDENTS.—For some time past much excitement has existed among some of the Southern medical students of this city, growing out, as has been alleged, of the Harper's Ferry movement. It had been vaguely rumored that a committee of thirty had been appointed early in the last month to obtain signatures to a pledge of secession; and on Tuesday morning, December twentieth, a final meeting was held at the Assembly Building rooms, preparatory to the exodus of the disaffected members of the classes of the different schools. The president of the meeting, we learn, was Mr. Lee, of Alabama, assisted by several vice-presidents and secretaries. Addresses were made on the occasion, among others, by Dr. F. E. Luckett and Dr. H. H. McGuire; and letters and telegraphic dispatches read from Governor Wise, of Virginia, and the deans of the medical colleges of Richmond, Charleston, Savannah, Augusta, New Orleans, and Nashville, tendering sympathy, and a cordial welcome to such secessionists as might feel inclined to resort to those institutions. The meeting is reported to have been conducted with great decorum. The time fixed upon for their departure from the city was Wednesday night, December twenty-first, with free passes provided by the Fredericksburg and Richmond Railroad, through Drs. Luckett and McGuire, over the whole route, and one thousand dollars, said to have been sent from Virginia, to defray incidental expenses.

The number of students that left has been variously estimated at from one to two hundred, a large majority of whom were matriculates of the Jefferson College. It is understood that they were joined at the depot by a small number of Southern students from the University of New York. The exodus was avowedly conducted by Dr. Luckett and Dr. McGuire, the former of whom had been intrusted with the railroad passes and the disbursement of the money. It is proper to add that these gentlemen had a large quizzing class, consisting of nearly two hundred Southern students, and generally known as the "Southern quizzing class." Of this class the great majority have left; and of the whole number of secessionists, one hundred, it is said, had previously been pledged to the medical college at Richmond.

The manner in which the secessionists were received at Richmond will appear by the following dispatch, copied from one of the daily journals of this city:—

*Reception of the Southern Medical Students at Richmond, Va.*

RICHMOND, VA., December 22.—The seceding medical students from Philadelphia arrived here to-day, and were received by the faculty and students of the medical college, the Governor's guard, and an immense throng of citizens. The procession marched to the Governor's mansion, where the students were addressed by Governor Wise, and afterwards by Professor Gibson, at the college. A dinner was then partaken of at the Columbia Hotel.

The students were received with great enthusiasm by our citizens, and as the procession passed through the streets the shouts of the men were deafening, while the ladies manifested their delight by the waving of their handkerchiefs.

The objects of the secessionists are best explained in the language of the preamble and resolutions adopted at the meeting at the Assembly Building rooms, on the twentieth instant:—

*Whereas*, We have left our homes and congregated in this city, with a view to prosecute our medical studies; and having become fully convinced that we have erred in taking this step; that our means should have been expended, and our protection afforded to the maintenance and advancement of institutions existing in our own sections, and fostered by our own people:—

*Resolved*, That in a body, or as many as approve of the act, we secede from the institutions in which we have severally matriculated, return to the South, and herein pledge ourselves to devote our future lives and best efforts to the protection of our common rights and the promotion of our common interests.

*Resolved*, That in taking this step, we disclaim any personal animosities, and deprecate any political agitation.

*Resolved*, That we tender our grateful acknowledgments and heartfelt thanks to the Hon. Henry A. Wise, Governor of Virginia; Dr. L. S. Joynes, Dean of the Virginia Medical College, at Richmond; Henry R. Frost, Dean of the Medical Department of the University of South Carolina; to President Robinson, of the Philadelphia, Wilmington, and Baltimore Railroad, and all others who have extended to us the substantial encouragement and aid so essential to the furtherance and successful accomplishment of our enterprise.

*Resolved*, That we extend a cordial invitation, and will cheerfully welcome in the South, any Northern student who will subscribe to the previous resolutions.

*Resolved*, That a copy of these proceedings be sent to all Northern medical colleges, for the benefit of Southern students who may have matriculated in them.

*Resolved*, That the Southern papers generally, be requested to publish the proceedings of this convention.

We have given the above statements of this proceeding as they have been communicated to us, as matters of interest to the medical profession of the United States in all time to come. As faithful journalists it is our duty to chronicle the event, and to express our profound regret at its occurrence. Various rumors are afloat in this city, both in the profession and in the community generally, as to the origin of this movement and those who played the chief part in its execution; but as we ourselves have no authentic data to guide us, we shall, for the present, forbear from any further comments.

